# Metformin Potentiates the Effects of Anlotinib in NSCLC *via* AMPK/mTOR and ROS-Mediated Signaling Pathways

**DOI:** 10.3389/fphar.2021.712181

**Published:** 2021-08-04

**Authors:** Zhongling Zhu, Teng Jiang, Huirong Suo, Shan Xu, Cai Zhang, Guoguang Ying, Zhao Yan

**Affiliations:** ^1^ Department of Clinical Pharmacology, Tianjin Medical University Cancer Institute and Hospital, National Clinical Research Center for Cancer, Key Laboratory of Cancer Prevention and Therapy, Tianjin’s Clinical Research Center for Cancer, Tianjin, China; ^2^ Department of Pharmacy, The Second Hospital of Tianjin Medical University, Tianjin, China; ^3^ Department of Tumor Cell Biology, Tianjin Medical University Cancer Institute and Hospital, National Clinical Research Center for Cancer, Key Laboratory of Cancer Prevention and Therapy, Tianjin’s Clinical Research Center for Cancer, Tianjin, China; ^4^ Department of Continuing Education and Science and Technology Service, China Anti-cancer Association, Tianjin, China

**Keywords:** non-small cell lung cancer, anlotinib, metformin, AMP-activated protein kinase, reactive oxygen species

## Abstract

Anlotinib is a novel multi-targeted tyrosine kinase inhibitor with activity against soft tissue sarcoma, small cell lung cancer, and non-small cell lung cancer (NSCLC). Potentiating the anticancer effect of anlotinib in combination strategies remains a clinical challenge. Metformin is an oral agent that is used as a first-line therapy for type 2 diabetes. Interesting, metformin also exerts broad anticancer effects through the activation of AMP-activated protein kinase (AMPK) and inhibition of mammalian target of rapamycin (mTOR). Here, we evaluated the possible synergistic effect of anlotinib and metformin in NSCLC cells. The results showed that metformin enhanced the antiproliferative effect of anlotinib. Moreover, anlotinib combined with metformin induced apoptosis and oxidative stress, which was associated with the activation of AMPK and inhibition of mTOR. Reactive oxygen species (ROS)- mediated p38/JNK MAPK and ERK signaling may be involved in the anticancer effects of this combination treatment. Our results show that metformin potentiates the efficacy of anlotinib *in vivo* by increasing the sensitivity of NSCLC cells to the drug. These data provide a potential rationale for the combination of anlotinib and metformin for the treatment of patients with NSCLC or other cancers.

## Introduction

Lung cancer is the leading cause of cancer-related death, accounting for over 1.7 million deaths per year worldwide (Bray et al., 2018). Approximately 85% of all lung cancers are non-small cell lung cancer (NSCLC). Most patients with NSCLC have advanced disease or local metastasis at diagnosis, and the 5-years overall survival rate is less than 20% (Hirsch et al., 2017). In recent years, checkpoint inhibitors and inhibitors of constitutively active EGFR, ALK, or ROS1 receptor tyrosine kinases (RTKs) have markedly improved tumor responses and clinical outcomes in patients with NSCLC (Hirsch et al., 2017; Assi et al., 2018).

Anlotinib is a novel, multitargeted tyrosine kinase inhibitor that has activity against a range of RTKs involved in vascularization and tumor progression, including VEGFR-1, -2, and -3; FGFR-1, -2, -3, and -4; c-kit; and PDGFR-α and -β (Shen et al., 2018; Gao et al., 2020). Several clinical trials have demonstrated that anlotinib is well tolerated and has promising efficacy in patients with solid tumors, including advanced NSCLC, soft tissue sarcoma, medullary thyroid carcinoma, esophageal squamous cell carcinoma, and metastatic renal cell carcinoma (Chi et al., 2018; Sun et al., 2018; Zhou et al., 2019; Ma et al., 2020; Wu et al., 2020; Huang et al., 2021). In phase III clinical trials, the median overall survival for patients with advanced NSCLC who progressed after treatment with at least two lines of prior systemic chemotherapy had increased by 3.3 months (Han et al., 2018). Based on these data, anlotinib was approved by the China National Medical Products Administration for third-line or further treatment of advanced NSCLC in 2018 (Syed, 2018).

The biguanide metformin is a first-line oral anti-diabetic drug. Several studies have shown that metformin inhibits cancer cell growth and induces both cell cycle arrest and apoptosis (Alimova et al., 2009; Dowling et al., 2011). Treatment with metformin has been reported to suppress the growth of tumor xenografts in nude mice (Wheaton et al., 2014). However, the mechanisms underlying these effects are poorly understood. It is known that metformin inhibits complex I of the mitochondrial electron transport chain (Fontaine, 2018), resulting in an increase in the intracellular AMP/ATP ratio and indirect activation of AMP-activated protein kinase (AMPK). AMPK activation promotes metabolic flexibility and net ATP conservation through multiple mechanisms, including activation of catabolic pathways, inhibition of anabolic processes that consume ATP, induction of autophagy, and maintenance of NADPH homeostasis to buffer reactive oxygen species (ROS). Retaining AMPK activity may protect tumor cells from bioenergetic catastrophe and provide them with a selective growth advantage under stress. Conversely, AMPK activation can inhibit mTOR signaling, leading to decreased HIFα-driven metabolism of glucose and glutamine (Faubert et al., 2015). Metformin can also exert anti-tumor effects through AMPK-independent pathways (Kalender et al., 2010).

Metformin was previously reported to increase the sensitivity of cancer cells to targeted therapies and chemotherapies (Zhang and Guo, 2016; Deng et al., 2019). Multiple combinations of metformin with targeted agents, such as gefitinib, trastuzumab, and temsirolimus, are currently being tested in phase I/II clinical trials (Khawaja et al., 2016; Zhang and Guo, 2016; Martin-Castillo et al., 2018). Here, we report the synergistic effect of anlotinib in combination with metformin *in vitro* and *in vivo*. We observed AMPK activation and inhibition of the downstream mTOR pathway, which may partly explain the synergistic cytotoxic effect. In addition, ROS-mediated p38/JNK MAPK and ERK signaling may be involved in the anticancer effect of the combination.

## Materials and Methods

### Reagents

Anlotinib was kindly provided by Chia Tai Tian Qing Pharmaceutical Group Co., Ltd (Nanjing, China). Metformin, 3- (4,5-dimethylthiazol-2-yl)-2,5-diphenyltetrazolium bromide (MTT), methanol, crystal violet, and phosphate-buffered saline (PBS) were purchased from Solarbio Bioscience & Technology Co. Ltd (Beijing, China). Hoechst 33342, propidium iodide (PI), 2,7-dichlorodihydrofluorescein diacetate (DCFH-DA), and SDS lysis buffer were obtained from Beyotime Biotechnology Co., Ltd (Shanghai, China). Dulbecco’s modified Eagle’s medium (DMEM), fetal bovine serum (FBS), trypsin, and penicillin/streptomycin were purchased from Invitrogen (Carlsbad, CA, United States).

### Cell Culture

The human lung cell lines A549 and H460 were obtained from American Type Culture Collection (Manassas, VA, United States) and were authenticated *via* DNA sequencing on an ABI 3730xl genetic analyzer. The cells were grown in DMEM supplemented with 10% heat-inactivated FBS, 100 units/ml penicillin, and 100 μg/ml streptomycin in a humidified atmosphere with 5% CO_2_ at 37°C.

### MTT Cell Viability Assay

Cells were seeded in 96-well culture plates at a density of 1–3 × 10^3^ cells/well. After 24 h, various concentrations of anlotinib (range, 0–20 μmol/L), metformin (range, 0–20 mmol/L), or both were added to the cells. After 72 h of incubation, 5 mg/ml MTT was added to each well and incubated for 4 h. The supernatants were carefully aspirated and the formazan crystals were dissolved in DMSO. Absorbance was recorded at 570 nm using a microplate reader.

### Colony Formation Assay

For clonogenic survival studies, 300 cells were seeded in 12-well plates and exposed to different treatments for 48 h. After 10–14 days of incubation, the colonies were fixed in cold methanol for 6 min and then stained with 1% crystal violet solution for 30 min. Colonies containing more than 50 cells were counted. Percent colony formation was calculated by comparison to that in untreated cultures, which was set to 100%. Thus, the percent colony formation of treated cells was calculated as follows: colony formation by treated cells/colony formation by untreated cells × 100.

### Apoptosis Assay

The apoptosis assay was performed using Hoechst 33342/PI fluorescence double staining. Briefly, A549 and H460 cells were seeded at approximately 50% confluence in six-well cell culture plates. Thereafter, cells were incubated with anlotinib (10 μM), metformin (10 mM), or both for 24 h. Finally, the treated cells were stained with Hoechst 33342 (10 μg/ml) and PI (5 μg/ml) at 37°C for 15 min and then imaged using fluorescence microscopy.

### Western Blot Analysis

Cells were washed with ice-cold PBS and lysed with SDS lysis buffer. The protein concentration in the lysates was determined using BCA reagent (Pierce, Rockford, IL, United States). The proteins were separated *via* SDS-polyacrylamide gel electrophoresis and then electrotransferred onto PVDF membranes (Millipore, Bedford, MA, United States). The membranes were blocked with 5% nonfat milk and incubated overnight at 4°C with primary antibodies against AMPK (#5832), phospho-AMPK (Thr172) (#2535), mTOR (#2983), phospho-mTOR (Ser2448) (#5536), acetyl-CoA carboxylase (ACC) (#3676), phospho-ACC (Ser79) (#3661), hypoxia-inducible factor 1α (HIF1α) (#36169), extracellular signal-regulated kinase (ERK1/2) (#4695), phospho-ERK1/2 (Thr202/Tyr204) (#4370), c-Jun NH 2-terminal kinase (JNK) (#9252), phospho-JNK (Thr183/Tyr185) (#4668), p38 (#8690), phospho-p38 (#4511) (Thr180/Tyr182), Bax (#5023), Bcl-2(#2870), cleaved caspase-3 (#9664), and cleaved PARP (#5625), which were purchased from Cell Signaling Technology (Danvers, MA, USA).Antibodies against *β*-actin (sc-47778) were purchased from Santa Cruz Biotechnology (Santa Cruz, CA, United States). After washing, the membranes were incubated with IRDye-conjugated anti-rabbit or anti-mouse IgG antibodies (LI-COR Biosciences, Lincoln, NE, United States). The proteins were visualized using an Odyssey LI-COR infrared imaging system.

### ROS Staining

Intracellular hydrogen peroxide was detected using a DCFH-DA fluorescent probe. Cells were cultured in six-well plates and treated as indicated. Cells were washed twice with PBS and then incubated with 10 μM DCFH-DA and 10 μg/ml Hoechst 33342 at 37°C for 30 min and then imaged using fluorescence microscopy.

### Determination of Intracellular ATP and the NADP^+^/NADPH Ratio

Relative intracellular ATP levels and the NADP^+^/NADPH ratio were determined using assay kits (Beyotime Biotechnology, Jiangsu, China) according to the manufacturer’s instructions. Briefly, cells were cultured in six-well plates and treated as indicated. At harvest, the cells were washed twice with PBS and lysed in lysis buffer. After centrifugation at 12,000 × *g* at 4°C for 5 min, the supernatant was collected. Intracellular ATP levels were determined using a luminescent plate reader. The protein concentration was quantified using BCA reagent to normalize protein levels for calculating ATP content.

To determine the NADP^+^/NADPH ratio, the cells were washed twice with PBS and lysed in NADP extraction buffer. Following centrifugation at 12,000 × *g* at 4°C for 10 min, the supernatants were collected and analyzed to quantify the NADP^+^/NADPH ratio according to the manufacturer’s instructions.

### Mouse Xenografts *in vivo*

Four-week-old female BALB/c nude mice were purchased from Beijing HFK Bioscience Co., Ltd (Beijing, China). The animals were maintained under controlled environmental conditions: 22–28°C, 60–70% relative humidity, and a 12 h dark/light cycle with water ad libitum. A549 cells (3 × 10^6^ cells) were intravenously injected into the left hind flanks of nude mice (n = 6 mice per group). Tumor volume was calculated using formula *V* = (*a* × *b* ^2^)/2, where *a* is the tumor length and *b* is the tumor width. When the tumor volume reached approximately 100 mm^3^, the mice were randomly assigned to the control [treated with vehicle (sterile PBS)], anlotinib (0.75 mg/kg), metformin (250 mg/kg), or combination (anlotinib plus metformin) groups. Anlotinib and metformin were intragastrically administered daily for 28 consecutive days. Tumor growth was monitored and measured twice per week using a Vernier caliper. Tumors were removed from the mice after 28 days of treatment. The relative tumor volume (RTV) was calculated as the ratio of the tumor volume at time *t* to the tumor volume at the start of treatment. Inhibition rates are expressed as the ratio of the RTV of the treatment group (TRTV) to the RTV of the control group (CRTV) by dividing the RTV of each treatment group by the RTV of the control group, and then multiplying the quotient by 100 (TRTV/CRTV%). All protocols were approved by the Laboratory Animal Ethics Committee of Tianjin Medical University Cancer Institute and Hospital.

### Statistical Analysis

In the xenograft experiment, randomization was performed using a computer-generated sequence of random numbers. In other experiments, randomization was not performed. Data are presented as the mean ± SE from three independent experiments. Data were analyzed using GraphPad Prism 5.01. Mean values were compared using the unpaired Student’s *t*-test. Normality distribution was assessed using the Shapiro-Wilk test. The combination index (CI) was calculated using CompuSyn software (Biosoft, Cambridge, United Kingdom). Statistical significance was set at *p* < 0.05 (*) or *p* < 0.01 (**).

## Results

### Metformin Potentiates the Antiproliferative Effect of Anlotinib in NSCLC Cells

**FIGURE 1:**
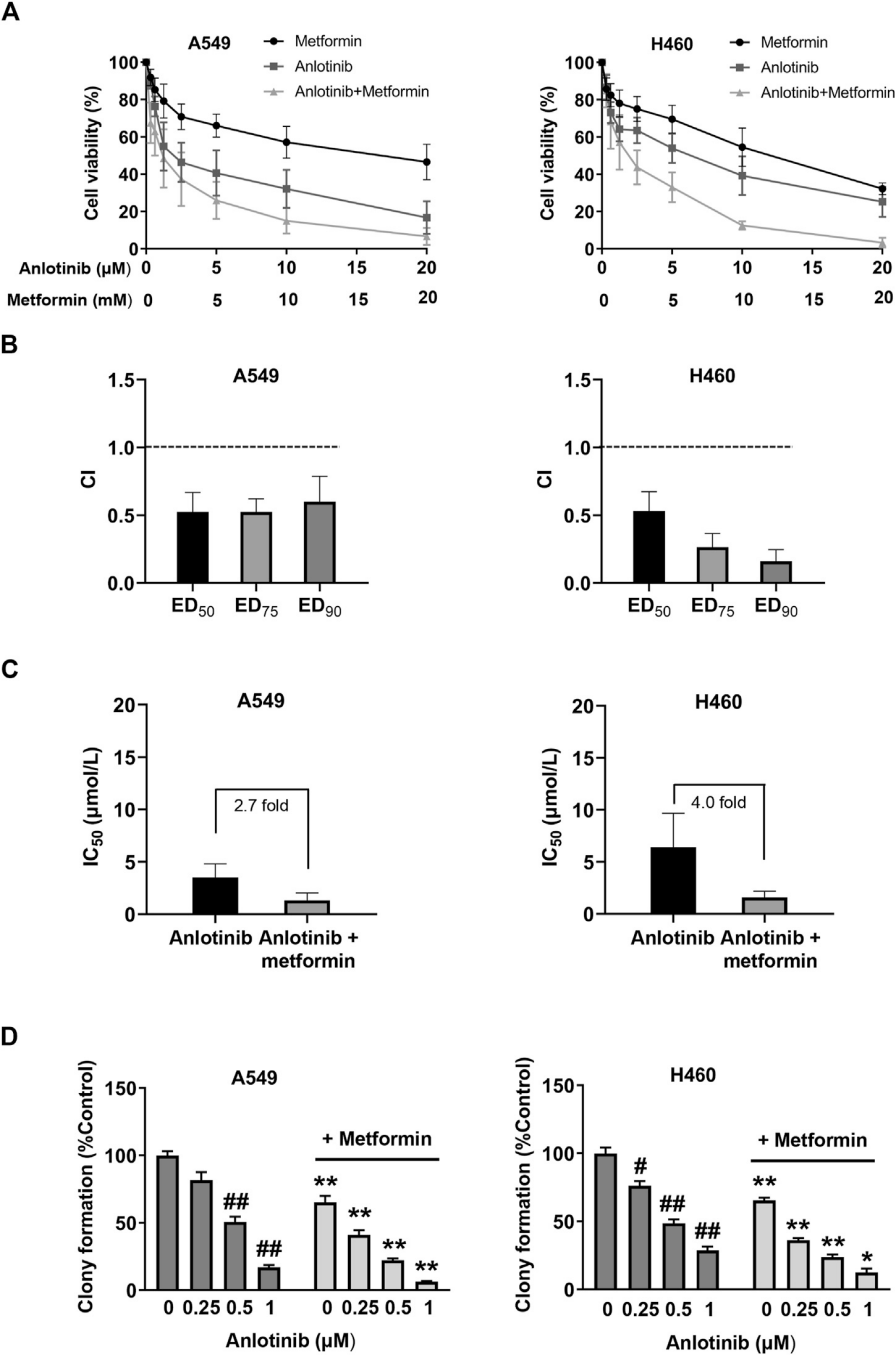
Metformin enhances the cytotoxicity of anlotinib in non-small cell lung cancer (NSCLC) cells **(A)** A549 and H460 NSCLC cells were treated with anlotinib (range, 0–20 μmol/L), metformin (range, 0–20 mmol/L), or both for 3 days. Cell viability (%) was determined using the MTT assay **(B)** The CI values were determined for effective dose (ED) ED_50_, ED_75_, and ED_90_. Columns represent data from triplicate analyses ±SE **(C)** The IC_50_ of anlotinib in A549 and H460 cells was reduced by the addition of metformin **(D)** A549 and H460 cells were exposed to the indicated concentrations of anlotinib alone or anlotinib combined with metformin (0.5 mM) for 48 h. Colony-forming efficiency was determined 10–14 days later. Data are presented as means ± SE from three independent experiments. Statistical significance was analyzed using unpaired Student’s *t*-test; *p < 0.05, **p < 0.01 compared with anlotinib alone; #*p* < 0.05, ##*p* < 0.01 compared with the untreated control group.

To examine the potential synergistic effect of anlotinib and metformin on cancer cell proliferation, we studied this drug combination in the NSCLC cell lines A549 and H460 using MTT and colony formation assays. As shown in Figure 1A, although each agent alone inhibited the proliferation of A549 and H46 cells, the combination had the strongest antiproliferative effect. The CI values were calculated using cytotoxicity data from the MTT assay. The results revealed that the CI values were less than one in both cell lines (Figure 1B). A CI value < 1 indicates drug synergism. The addition of metformin reduced the half-maximal inhibitory concentration of anlotinib by 2.7-fold in A549 cells and by 4.0-fold in H460 cells (Figure 1C). We also tested this combination in a 14-days colony formation assay. Similarly, combined treatment with anlotinib and metformin synergistically suppressed colony formation in both A549 and H460 cells (Figure 1D). These data indicate that the antiproliferative effects of anlotinib and metformin are strongly synergistic in A549 and H460 cells.

### Metformin Enhances the Efficacy of Anlotinib in Tumor Xenografts

**FIGURE 2:**
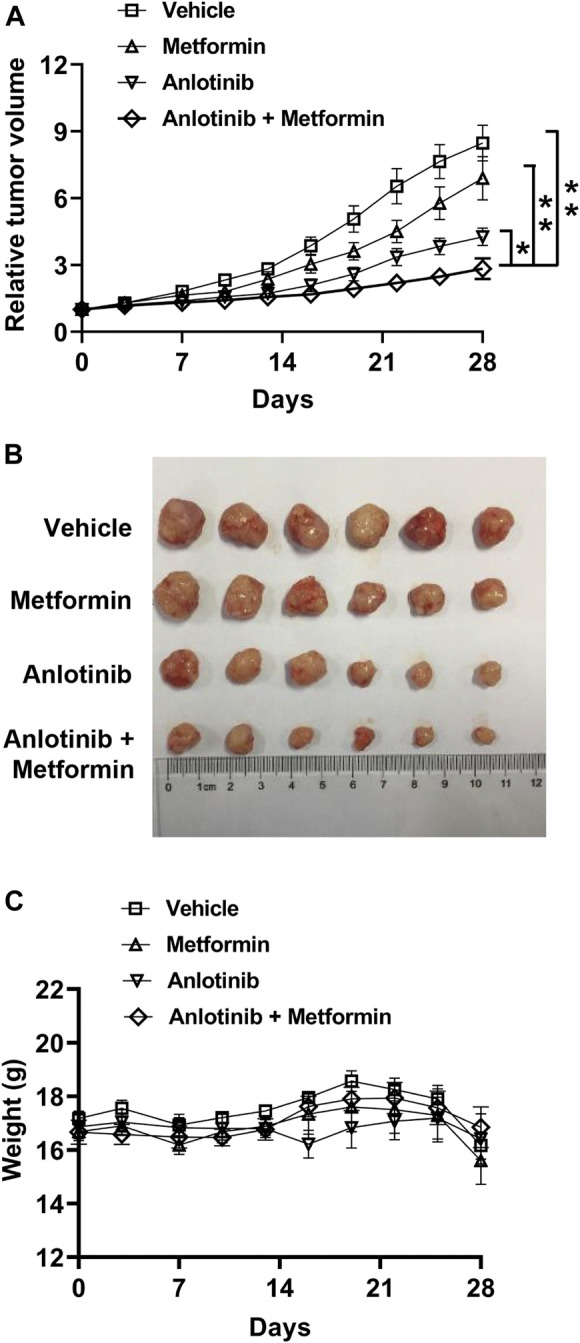
Metformin potentiates the efficacy of anlotinib in a xenograft model **(A)** Mice with H460 xenografts were randomly divided into four groups and treated for 28 consecutive days with vehicle (squares), anlotinib (triangles), metformin (inverted triangles), or both anlotinib and metformin (diamonds) as described in the *Materials and Methods* **(B)** The sizes of the xenografts at the end of experiment **(C)** Body weight was measured every 3 days. Data are presented as means ± SE (n = 6 per group). Statistical significance was analyzed using the unpaired Student’s *t*-test; *p < 0.05, **p < 0.01.

We evaluated whether combination treatment with metformin enhanced the antitumor effects of anlotinib using A549 xenografts. Nude mice bearing A549 xenografts were randomized and treated either with anlotinib (0.75 mg/kg), metformin (250 mg/kg), or both. Although anlotinib and metformin as monotherapies decreased tumor growth when compared with the control, the combination treatment potentiated the antitumor effects of each single treatment (Figures 2A,B), indicating that the cytotoxicity of anlotinib in the xenograft model was enhanced by the addition of metformin. No significant weight loss was observed in any of the treatment groups, suggesting that the toxicity of the combination was acceptable (Figure 2C).

### Anlotinib in Combination with Metformin Induces Cell Apoptosis

**FIGURE 3:**
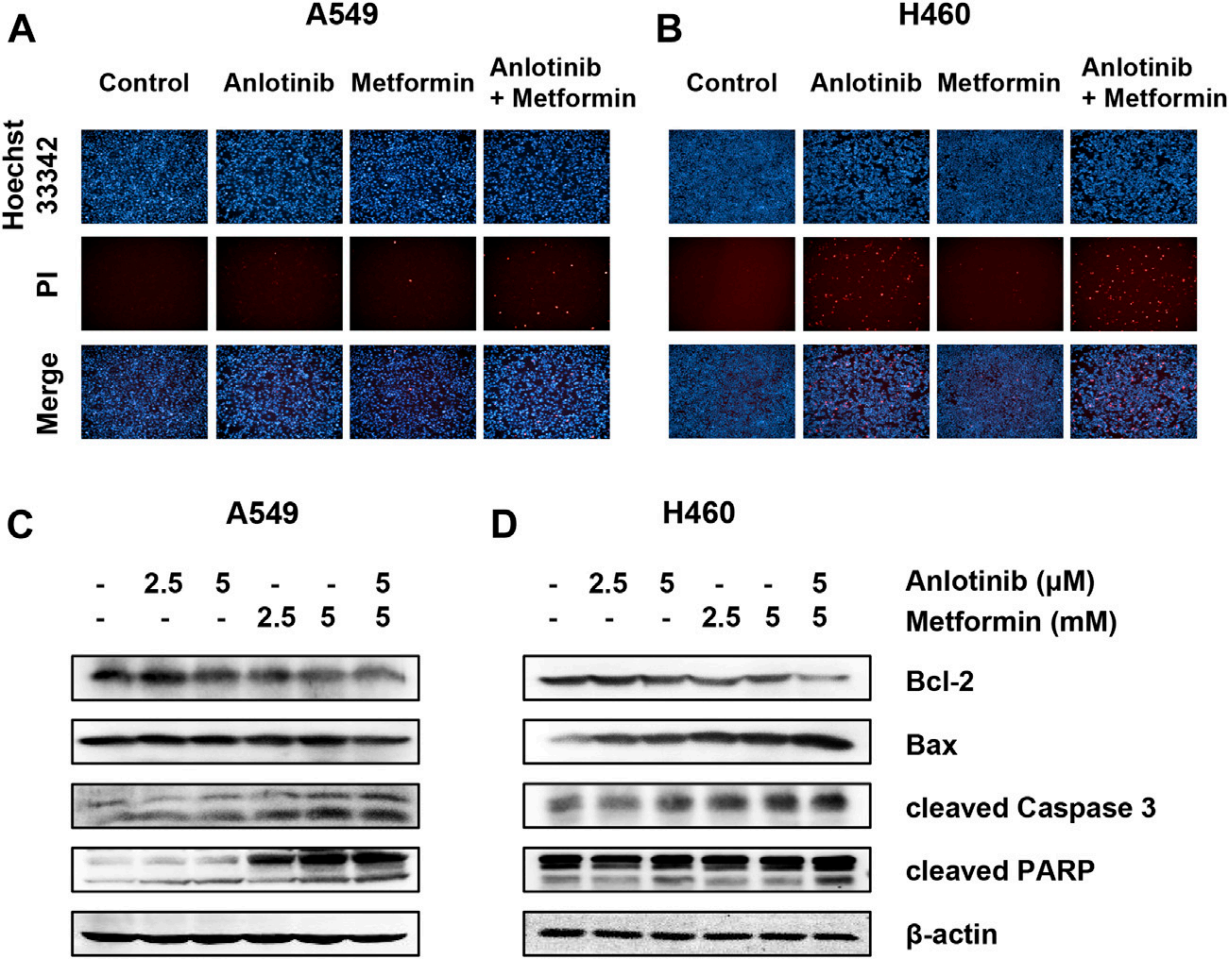
Anlotinib with metformin synergistically induces apoptosis. A549 **(A)** and H460 **(B)** cells were incubated with anlotinib (10 μM), metformin (10 mM), or both for 24 h. After Hoechst 33342 (bright blue color) and PI (red color) double staining, the morphological changes in cells undergoing apoptosis and necrosis were observed under a fluorescence microscope. A549 **(C)** and H460 **(D)** cells were treated with the indicated concentrations of anlotinib, metformin, or both for 24 h. Cell lysates were immunoblotted with antibodies against Bcl-2, Bax, cleaved caspase 3, and PARP.

We examined the effects of anlotinib and metformin on cell apoptosis *via* Hoechst 33342/PI double staining under a fluorescence microscope. As shown in Figures 3A,B, higher numbers of apoptotic and necrotic cells, with condensation of nuclear chromatin and fragmentation, were detected in cells treated with the combination of anlotinib and metformin compared to cells treated with monotherapy. We then determined the expression levels of the apoptosis-related proteins Bcl-2, Bax, caspase 3, and PARP using western blot analysis. The level of Bax protein was substantially increased in response to combination treatment, whereas the expression of Bcl-2 was reduced. We also found that the combination treatment induced caspase 3 and PARP cleavage to an even greater extent (Figures 3C,D). These results suggested that the combination treatment likely induced the Bcl-2/Bax-caspase signaling pathway.

### The Synergistic Effect of Anlotinib and Metformin is Mediated by AMPK Activation and mTOR Inhibition

**FIGURE 4:**
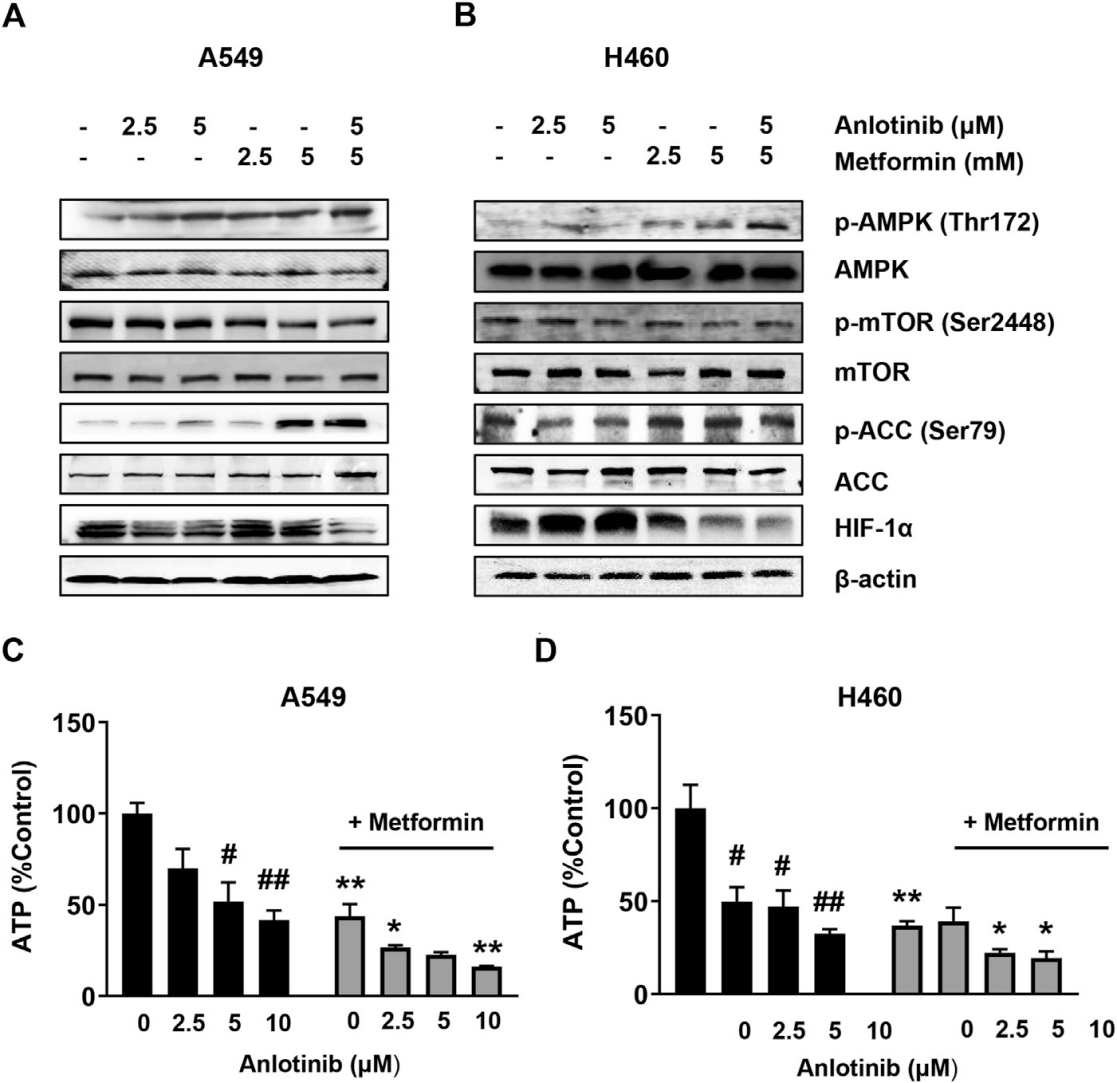
Anlotinib in combination with metformin promotes the activation of AMPK and inhibition of mTOR. A549 **(A)** and H460 **(B)** cells were treated with the indicated concentrations of anlotinib, metformin, or both for 24 h. Cell lysates were harvested and immunoblotted with the indicated antibodies. A549 **(C)** and H460 **(D)** were exposed to the indicated concentrations of anlotinib alone or anlotinib and metformin (5 mM) for 24 h. Relative intracellular ATP levels were determined using an assay kit according to the manufacturer’s instructions. Data are presented as means ± SE from three independent experiments. Statistical significance was analyzed using unpaired Student’s *t*-test; *p < 0.05, **p < 0.01 compared with anlotinib alone; #*p* < 0.05, ##*p* < 0.01 compared with the untreated control group.

To clarify the mechanisms underlying the antiproliferative effects of the combination treatment, we studied the effects of the combined treatment on the AMPK and mTOR pathways. Our results showed that anlotinib treatment alone induced phosphorylation of AMPKα at Thr-172. Importantly, AMPK activation increased significantly when anlotinib was combined with metformin (Figure 4). AMPK activation has been shown to reduce cell proliferation, at least in part, by inhibiting mTOR signaling. We found that the combination treatment had a synergistic effect on the suppression of mTOR phosphorylation. It has been reported that acetyl-coA carboxylase (ACC), which plays an important role in the biosynthesis and oxidation of fatty acids, is a downstream substrate of AMPK signaling. Indeed, we found that phosphorylation of ACC at Ser79 was markedly increased when cells were treated with both anlotinib and metformin (Figures 4A,B). Previous studies indicated that metformin might inhibit tumor growth by inhibiting complex I of the respiratory chain and decreasing ATP production. Interestingly, as shown in Figures 4C,D the generation of intracellular ATP in A549 and H460 cells was markedly inhibited by exposure to anlotinib in a concentration-dependent manner. In addition, the combination treatment resulted in a lower intracellular ATP levels, suggesting that anlotinib and metformin synergistically inhibited the production of intracellular ATP. HIF-1 transcriptionally promotes anaerobic glycolysis, leading to increased ATP production. Therefore, we next examined HIF-1α expression in treated cells and found that the expression of HIF-1α was markedly inhibited in both A549 and H460 cells exposed to the combination treatment under normoxic conditions (Figures 4A,B). These data suggest that the synergistic effect of anlotinib and metformin is related to regulation of the AMPK/mTOR signaling pathway.

### The Combination of Anlotinib and Metformin Promotes Oxidative Stress

**FIGURE 5:**
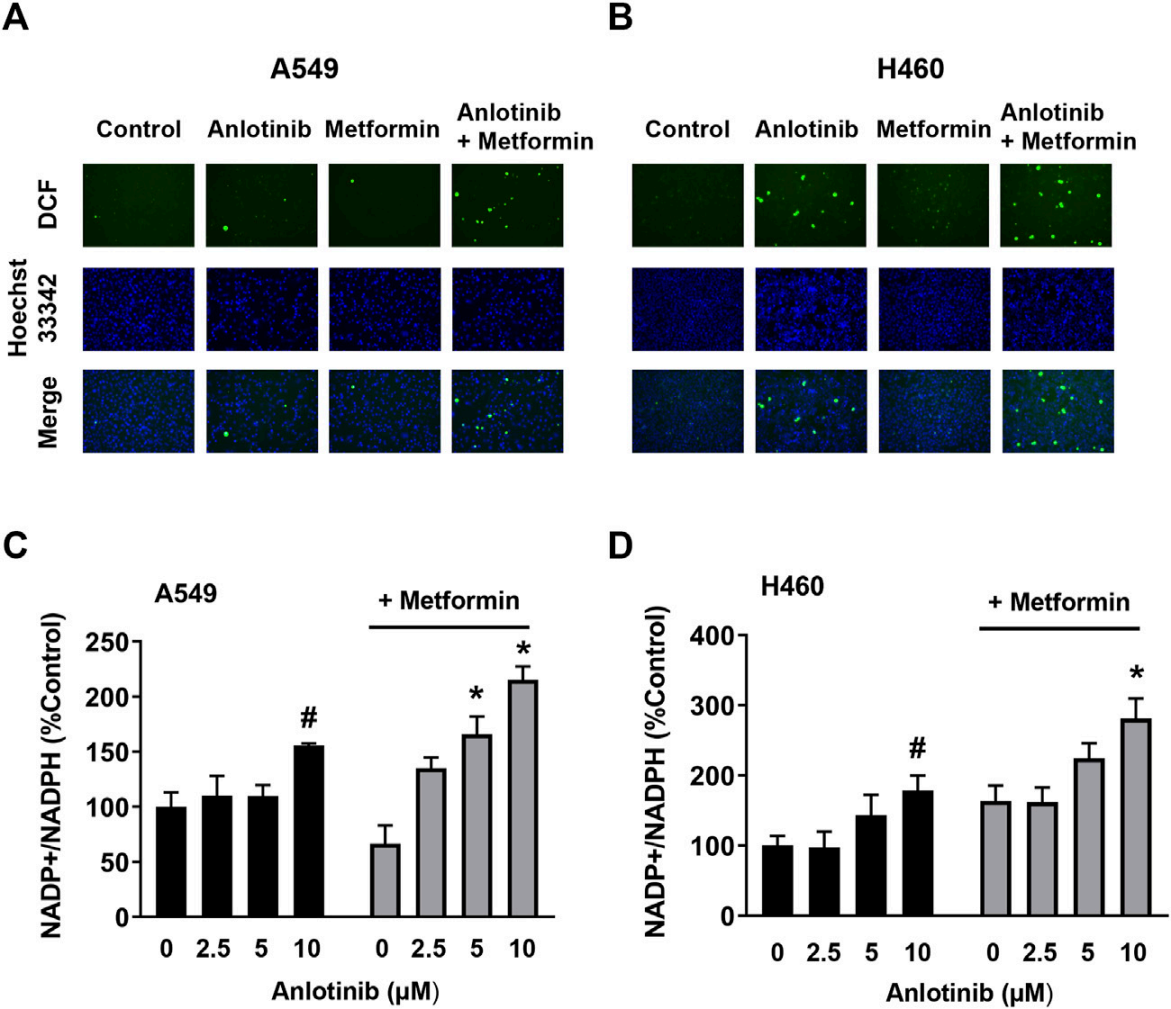
Anlotinib combined with metformin increased intracellular ROS levels and the NADP^+^/NADPH ratio. A549 **(A)** and H460 **(B)** cells were incubated with anlotinib (5 μM), metformin (5 mM), or both for 24 h. After DCF (green color) and Hoechst 33342 (bright blue color) double staining, cellular DCF fluorescence was observed under a fluorescence microscope. A549 **(C)** and H460 **(D)** cells were exposed to the indicated concentrations of anlotinib or anlotinib and metformin (5 mM) for 24 h. The NADP^+^/NADPH ratio was measured in A549 and H460 cells using an assay kit, as described in the *Materials and Methods*. Data are presented as means ± SE from three independent experiments. Statistical significance was analyzed using unpaired Student’s *t*-test; *p < 0.05 compared with anlotinib alone; #*p* < 0.05 compared with the untreated control group.

ROS plays a crucial role in cell apoptosis signaling pathways. Therefore, we examined whether ROS is involved in the cytotoxic effects of anlotinib. The changes in total ROS production were estimated using a cell-permeable probe DCFH-DA. The fluorescence signals for DCF were markedly enhanced in cells treated with anlotinib and metformin compared to the signals in cells treated with anlotinib or metformin alone (Figures 5A,B). Intracellular NADP^+^/NADPH is believed to be a critical redox couple against oxidative stress. Anlotinib significantly increased the ratio of intracellular NADP^+^/NADPH in both cell types. The effect of anlotinib on the NADP^+^/NADPH ratio was potentiated in the presence of metformin, indicating that the combination treatment regulated intracellular redox homeostasis and promoted switching to the oxidative state (Figures 5C,D). These results indicate that the combination treatment induced oxidative stress in A549 and H460 cells.

### Anlotinib Combined with Metformin Stimulates the Kinase Activities of p38, JNK, and ERK1/2 Kinases

**FIGURE 6:**
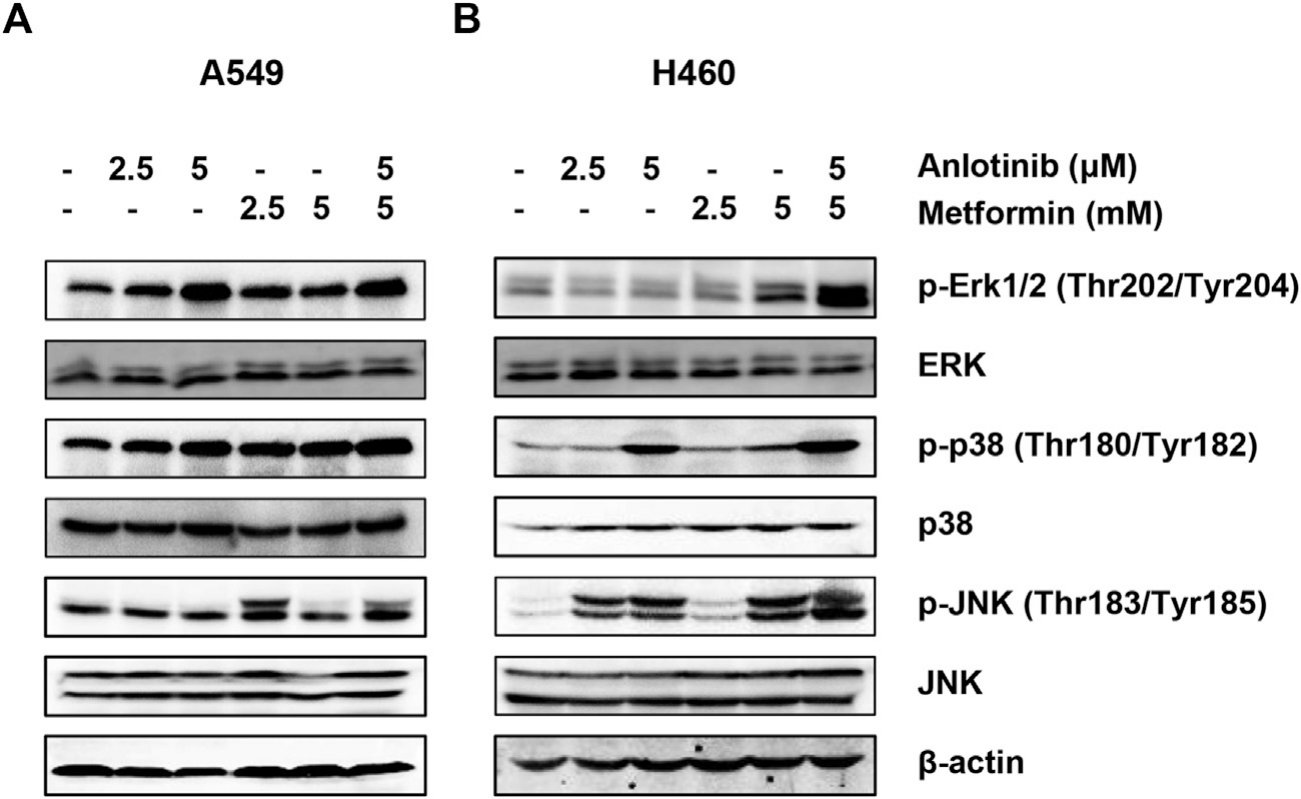
Anlotinib combined with metformin increases the phosphorylation of ERK1/2, p38, and JNK. A549 **(A)** and H460 **(B)** cells were incubated with the indicated concentrations of anlotinib, metformin, or both for 24 h. Cell lysates were immunoblotted with antibodies against pERK1/2 and ERK1/2, p-p38 and p38, and pJNK and JNK.

ROS play a critical role in cell death *via* regulation of the mitogen-activated protein kinase (MAPK) family. Here, we studied the effects of anlotinib and metformin both alone and in combination on the kinase activities of ERK1/2, p38, and JNK. Our results showed that the combination treatment exerted a synergistic effect on p38, JNK, and ERK1/2 activation (Figure 6). Thus, these results indicate that anlotinib in combination with metformin enhances the phosphorylation of p38, JNK, and ERK1/2, which may be mediated by ROS.

## Discussion

The anticancer effect of anlotinib has been reported to be associated with its function as a multikinase inhibitor in angiogenic signaling pathways. Anlotinibalso suppresses tumor growth by blocking c-Kit, RET, Aurora-B, c-FMS, and DDR1 (Sun et al., 2016). In this study, we revealed that metformin could augment the cytotoxic effects of anlotinib. The enhanced synergistic effect of anlotinib and metformin inhibited the proliferation of NSCLC cells both *in vitro* and *in vivo*. The concentrations of anlotinib and metformin administrated in our *in vitro* and *in vivo* experiments are equal to concentrations administrated in previous studies (Chen et al., 2012; Xie et al., 2020), though higher than the therapeutic doses in humans. Low concentrations of metformin (typically 0.1–0.3 mM) selectively inhibited cancer stem cells, but these low doses had little effect on the proliferation of cancer cells (Ben et al., 2010). The combination treatment increased PARP1 cleavage, caspase-3 cleavage, and the Bax/Bcl-2 ratio, suggesting that the combination treatment triggered apoptosis, possibly mediated by the mitochondrial-dependent pathway.

Previous studies have mostly focused on the effects of anlotinib on angiogenesis and proliferation (Song F. et al., 2020). Comparatively, little attention has been paid to the effects of anlotinib on energy metabolism. In this study, we found that anlotinib significantly decreased the ATP content in NSCLC cells. It is known that decreased ATP can activate AMPK, which inhibits growth by blocking the mTOR pathway (Faubert et al., 2015). Metformin has been shown to reduce cell proliferation through the activation of AMPK and inactivation of mTOR signaling (Rocha et al., 2011; Storozhuk et al., 2013). In our study, we found that anlotinib activated AMPK, downregulated mTOR phosphorylation, and induced apoptosis. Importantly, metformin, as an indirect AMPK activator, potentiates the effects of anlotinib on the AMPK and mTOR signaling pathways. In agreement with our findings, Groenendijk et al. showed that sorafenib synergizes with metformin in NSCLC through the activation of the AMPK pathway (Groenendijk et al., 2015).

As signaling molecules, ROS play a crucial role in cell death signal transduction pathways. Excessive ROS can cause damage to biomacromolecules and promote autophagy, apoptosis, or necrosis (Wong et al., 2010). The NADP^+^/NADPH redox couple is involved in buffering ROS and sustaining antioxidant defenses (Aon et al., 2010). Recently, Yang et al. reported that anlotinib can directly inhibit the proliferation of and induce apoptosis in pancreatic cancer cells through ROS-activated ER stress *via* PERK/p-eIF2α/ATF4 (Yang L. et al., 2020). Similarly, we found that anlotinib or metformin increased ROS production and the NADP^+^/NADPH ratio in NSCLC cells, indicating that anlotinib or metformin can disrupt intracellular redox homeostasis and induce oxidative stress. Moreover, the combination treatment stimulated ROS generation to an even greater extent. Growing evidence has shown that members of the MAPK family, including p38 MAPK, JNK, and ERK, are critically involved inthe oxidative stress response (El-Najjar et al., 2010). The p38 MAPK and JNK pathways are related to apoptosis, yet activation of ERK is also related to cell survival (Zhang et al., 2019). However, many studies have shown that activation of ERK can promote cell death *via* apoptotic pathways and cell cycle arrest (Wang et al., 2000; Tang et al., 2002; Song Y. et al., 2020). These effects require sustained activation of ERK in specific subcellular compartments (Wang et al., 2000). In our study, we observed that anlotinib increased the phosphorylation of ERK1/2, p38, and JNK, and these increases in phosphorylation were greatest when cells were treated with both anlotinib and metformin. These data suggest that anlotinib-induced apoptosis may be the result of elevated intracellular ROS, which may function as upstream regulators of the p38/JNK MAPK and ERK pathways. In contrast, other studies have shown that anlotinib attenuates ERK activation in diverse cancer cells (Yang Q. et al., 2020; Hu et al., 2020; Lian et al., 2020). Cagnol et al. reported that ERK activity depends on the presence of ROS (Cagnol and Chambard, 2010). Differences in intracellular ROS levels and patterns of ROS accumulation may contribute to this inconsistency.

Thus, several mechanisms may contribute to the synergistic anticancer effect of anlotinib and metformin. The first involves decreased ATP-induced AMPK activation and mTOR inhibition. Additionally, ROS-mediated induction of p38/JNK MAPK and ERK signaling may be involved.

## Conclusion

Metformin increases the sensitivity of NSCLC cells to anlotinib both *in vitro* and *in vivo*, providing a potential rationale for the combination of anlotinib with metformin for patients with NSCLC or other cancers.

## Data Availability

The original contributions presented in the study are included in the article/supplementary materials, further inquiries can be directed to the corresponding author.

## References

[B1] Alimova I. N. Liu B. Fan Z. Edgerton S. M. Dillon T. Lind S. E. (2009). Metformin Inhibits Breast Cancer Cell Growth, colony Formation and Induces Cell Cycle Arrest *In Vitro* . Cell Cycle 8 (6), 909–915. 10.4161/cc.8.6.7933 19221498

[B2] Aon M. A. Cortassa S. O'Rourke B. (2010). Redox-optimized ROS Balance: a Unifying Hypothesis. Biochim. Biophys. Acta (Bba) - Bioenerg. 1797 (6-7), 865–877. 10.1016/j.bbabio.2010.02.016 PMC2891851 20175987

[B3] Assi H. I. Kamphorst A. O. Moukalled N. M. Ramalingam S. S. (2018). Immune Checkpoint Inhibitors in Advanced Non-small Cell Lung Cancer. Cancer 124 (2), 248–261. 10.1002/cncr.31105 29211297

[B4] Ben Sahra I. Le Marchand-Brustel Y. Tanti J. F. Bost F. (2010). Metformin in Cancer Therapy: a New Perspective for an Old Antidiabetic Drug?. Mol. Cancer Ther. 9 (5), 1092–1099. 10.1158/1535-7163.MCT-09-1186 20442309

[B5] Bray F. Ferlay J. Soerjomataram I. Siegel R. L. Torre L. A. Jemal A. (2018). Global Cancer Statistics 2018: GLOBOCAN Estimates of Incidence and Mortality Worldwide for 36 Cancers in 185 Countries. CA: A Cancer J. Clinicians 68 (6), 394–424. 10.3322/caac.21492 30207593

[B6] Cagnol S. Chambard J. C. (2010). ERK and Cell Death: Mechanisms of ERK-Induced Cell Death - Apoptosis, Autophagy and Senescence. FEBS J. 277 (1), 2–21. 10.1111/j.1742-4658.2009.07366.x 19843174

[B7] Chen G. Xu S. Renko K. Derwahl M. (2012). Metformin Inhibits Growth of Thyroid Carcinoma Cells, Suppresses Self-Renewal of Derived Cancer Stem Cells, and Potentiates the Effect of Chemotherapeutic Agents. J. Clin. Endocrinol. Metab. 97 (4), E510–E520. 10.1210/jc.2011-1754 22278418

[B8] Chi Y. Fang Z. Hong X. Yao Y. Sun P. Wang G. (2018). Safety and Efficacy of Anlotinib, a Multikinase Angiogenesis Inhibitor, in Patients with Refractory Metastatic Soft-Tissue Sarcoma. Clin. Cancer Res. 24, 5233–5238. 10.1158/1078-0432.CCR-17-3766 29895706

[B9] Deng J. Peng M. Wang Z. Zhou S. Xiao D. Deng J. (2019). Novel Application of Metformin Combined with Targeted Drugs on Anticancer Treatment. Cancer Sci. 110 (1), 23–30. 10.1111/cas.13849 30358009 PMC6317954

[B10] Dowling R. J. Goodwin P. J. Stambolic V. (2011). Understanding the Benefit of Metformin Use in Cancer Treatment. BMC Med. 9, 33. 10.1186/1741-7015-9-33 21470407 PMC3224599

[B11] El-Najjar N. Chatila M. Moukadem H. Vuorela H. Ocker M. Gandesiri M. (2010). Reactive Oxygen Species Mediate Thymoquinone-Induced Apoptosis and Activate ERK and JNK Signaling. Apoptosis 15 (2), 183–195. 10.1007/s10495-009-0421-z 19882352

[B12] Faubert B. Vincent E. E. Poffenberger M. C. Jones R. G. (2015). The AMP-Activated Protein Kinase (AMPK) and Cancer: Many Faces of a Metabolic Regulator. Cancer Lett. 356 (2), 165–170. 10.1016/j.canlet.2014.01.018 24486219

[B13] Fontaine E. (2018). Metformin-Induced Mitochondrial Complex I Inhibition: Facts, Uncertainties, and Consequences. Front. Endocrinol. 9, 753. 10.3389/fendo.2018.00753 PMC6304344 30619086

[B14] Gao Y. Liu P. Shi R. (2020). Anlotinib as a Molecular Targeted Therapy for Tumors (Review). Oncol. Lett. 20 (2), 1001–1014. 10.3892/ol.2020.11685 32724339 PMC7377159

[B15] Groenendijk F. H. Mellema W. W. van der Burg E. Schut E. Hauptmann M. Horlings H. M. (2015). Sorafenib Synergizes with Metformin in NSCLC through AMPK Pathway Activation. Int. J. Cancer 136 (6), 1434–1444. 10.1002/ijc.29113 25080865 PMC4312923

[B16] Han B. Li K. Wang Q. Zhang L. Shi J. Wang Z. (2018). Effect of Anlotinib as a Third-Line or Further Treatment on Overall Survival of Patients with Advanced Non-small Cell Lung Cancer. JAMA Oncol. 4 (11), 1569. 10.1001/jamaoncol.2018.3039 30098152 PMC6248083

[B17] Hirsch F. R. Scagliotti G. V. Mulshine J. L. Kwon R. Curran W. J. Wu Y.-L. (2017). Lung Cancer: Current Therapies and New Targeted Treatments. The Lancet 389 (10066), 299–311. 10.1016/S0140-6736(16)30958-8 27574741

[B18] Hu H. Liu Y. Tan S. Xie X. X. He J. Luo F. (2020). Anlotinib Exerts Anti-cancer Effects on KRAS-Mutated Lung Cancer Cell through Suppressing the MEK/ERK Pathway. Cmar 12, 3579–3587. 10.2147/CMAR.S243660 PMC7250708 32547195

[B19] Huang J. Xiao J. Fang W. Lu P. Fan Q. Shu Y. (2021). Anlotinib for Previously Treated Advanced or Metastatic Esophageal Squamous Cell Carcinoma: A Double‐blind Randomized Phase 2 Trial. Cancer Med. 10 (5), 1681–1689. 10.1002/cam4.3771 33586360 PMC7940231

[B20] Kalender A. Selvaraj A. Kim S. Y. Gulati P. Brûlé S. Viollet B. (2010). Metformin, Independent of AMPK, Inhibits mTORC1 in a Rag GTPase-dependent Manner. Cel. Metab. 11 (5), 390–401. 10.1016/j.cmet.2010.03.014 PMC3081779 20444419

[B21] Khawaja M. R. Nick A. M. Madhusudanannair V. Fu S. Hong D. Mcquinn L. M. (2016). Phase I Dose Escalation Study of Temsirolimus in Combination with Metformin in Patients with Advanced/refractory Cancers. Cancer Chemother. Pharmacol. 77 (5), 973–977. 10.1007/s00280-016-3009-7 27014780 PMC5978416

[B22] Lian Z. Du W. Zhang Y. Fu Y. Liu T. Wang A. (2020). Anlotinib Can Overcome Acquired Resistance to EGFR‐TKIs *via* FGFR1 Signaling in Non‐small Cell Lung Cancer without Harboring EGFR T790M Mutation. Thorac. Cancer 11 (7), 1934–1943. 10.1111/1759-7714.13485 32433828 PMC7327692

[B23] Ma J. Song Y. Shou J. Bai Y. Li H. Xie X. (2020). Anlotinib for Patients with Metastatic Renal Cell Carcinoma Previously Treated with One Vascular Endothelial Growth Factor Receptor-Tyrosine Kinase Inhibitor: A Phase 2 Trial. Front. Oncol. 10, 664. 10.3389/fonc.2020.00664 32457838 PMC7221023

[B24] Martin-Castillo B. Pernas S. Dorca J. Álvarez I. Martínez S. Pérez-Garcia J. M. (2018). A Phase 2 Trial of Neoadjuvant Metformin in Combination with Trastuzumab and Chemotherapy in Women with Early HER2-Positive Breast Cancer: the METTEN Study. Oncotarget 9 (86), 35687–35704. 10.18632/oncotarget.26286 30479698 PMC6235018

[B25] Rocha G. Z. Dias M. M. Ropelle E. R. Osório-Costa F. Rossato F. A. Vercesi A. E. (2011). Metformin Amplifies Chemotherapy-Induced AMPK Activation and Antitumoral Growth. Clin. Cancer Res. 17 (12), 3993–4005. 10.1158/1078-0432.CCR-10-2243 21543517

[B26] Shen G. Zheng F. Ren D. Du F. Dong Q. Wang Z. (2018). Anlotinib: a Novel Multi-Targeting Tyrosine Kinase Inhibitor in Clinical Development. J. Hematol. Oncol. 11 (1), 120. 10.1186/s13045-018-0664-7 30231931 PMC6146601

[B27] Song F. Hu B. Cheng J.-W. Sun Y.-F. Zhou K.-Q. Wang P.-X. (2020a). Anlotinib Suppresses Tumor Progression *via* Blocking the VEGFR2/PI3K/AKT cascade in Intrahepatic Cholangiocarcinoma. Cell Death Dis 11 (7), 573. 10.1038/s41419-020-02749-7 32709873 PMC7381674

[B28] Song Y. Sun X. Duan F. He C. Wu J. Huang X. (2020b). SYPL1 Inhibits Apoptosis in Pancreatic Ductal Adenocarcinoma *via* Suppression of ROS-Induced ERK Activation. Front. Oncol. 10, 1482. 10.3389/fonc.2020.01482 33042794 PMC7522464

[B29] Storozhuk Y. Hopmans S. N. Sanli T. Barron C. Tsiani E. Cutz J.-C. (2013). Metformin Inhibits Growth and Enhances Radiation Response of Non-small Cell Lung Cancer (NSCLC) through ATM and AMPK. Br. J. Cancer 108 (10), 2021–2032. 10.1038/bjc.2013.187 23632475 PMC3670487

[B30] Sun Y. Du F. Gao M. Ji Q. Li Z. Zhang Y. (2018). Anlotinib for the Treatment of Patients with Locally Advanced or Metastatic Medullary Thyroid Cancer. Thyroid 28 (11), 1455–1461. 10.1089/thy.2018.0022 30142994

[B31] Sun Y. Niu W. Du F. Du C. Li S. Wang J. (2016). Safety, Pharmacokinetics, and Antitumor Properties of Anlotinib, an Oral Multi-Target Tyrosine Kinase Inhibitor, in Patients with Advanced Refractory Solid Tumors. J. Hematol. Oncol. 9 (1), 105. 10.1186/s13045-016-0332-8 27716285 PMC5051080

[B32] Syed Y. Y. (2018). Anlotinib: First Global Approval. Drugs 78 (10), 1057–1062. 10.1007/s40265-018-0939-x 29943374

[B33] Tang D. Wu D. Hirao A. Lahti J. M. Liu L. Mazza B. (2002). ERK Activation Mediates Cell Cycle Arrest and Apoptosis after DNA Damage Independently of P53. J. Biol. Chem. 277 (15), 12710–12717. 10.1074/jbc.M111598200 11821415

[B34] Wang X. Martindale J. L. Holbrook N. J. (2000). Requirement for ERK Activation in Cisplatin-Induced Apoptosis. J. Biol. Chem. 275 (50), 39435–39443. 10.1074/jbc.M004583200 10993883

[B35] Wheaton W. W. Weinberg S. E. Hamanaka R. B. Soberanes S. Sullivan L. B. Anso E. (2014). Metformin Inhibits Mitochondrial Complex I of Cancer Cells to Reduce Tumorigenesis. eLife 3. 10.7554/eLife.02242 PMC4017650 24843020

[B36] Wong C. H. Iskandar K. B. Yadav S. K. Hirpara J. L. Loh T. Pervaiz S. (2010). Simultaneous Induction of Non-canonical Autophagy and Apoptosis in Cancer Cells by ROS-dependent ERK and JNK Activation. PLoS One 5 (4), e9996. 10.1371/journal.pone.0009996 20368806 PMC2848860

[B37] Wu D. Nie J. Hu W. Dai L. Zhang J. Chen X. (2020). A Phase II Study of Anlotinib in 45 Patients with Relapsed Small Cell Lung Cancer. Int. J. Cancer 147, 3453–3460. 10.1002/ijc.33161 32557583 PMC7689882

[B38] Xie J. Ye J. Cai Z. Luo Y. Zhu X. Deng Y. (2020). GPD1 Enhances the Anticancer Effects of Metformin by Synergistically Increasing Total Cellular Glycerol-3-Phosphate. Cancer Res. 80 (11), 2150–2162. 10.1158/0008-5472.CAN-19-2852 32179514

[B39] Yang L. Zhou X. Sun J. Lei Q. Wang Q. Pan D. (2020a). Reactive Oxygen Species Mediate Anlotinib-Induced Apoptosis *via* Activation of Endoplasmic Reticulum Stress in Pancreatic Cancer. Cel. Death Dis. 11 (9), 766. 10.1038/s41419-020-02938-4 PMC7499216 32943607

[B40] Yang Q. Ni L. Imani S. Xiang Z. Hai R. Ding R. (2020b). Anlotinib Suppresses Colorectal Cancer Proliferation and Angiogenesis *via* Inhibition of AKT/ERK Signaling Cascade. Cmar 12, 4937–4948. 10.2147/CMAR.S252181 PMC7321688 32606981

[B41] Zhang G. He J. Ye X. Zhu J. Hu X. Shen M. (2019). β-Thujaplicin Induces Autophagic Cell Death, Apoptosis, and Cell Cycle Arrest through ROS-Mediated Akt and P38/ERK MAPK Signaling in Human Hepatocellular Carcinoma. Cel. Death Dis. 10 (4), 255. 10.1038/s41419-019-1492-6 PMC6420571 30874538

[B42] Zhang H. H. Guo X. L. (2016). Combinational Strategies of Metformin and Chemotherapy in Cancers. Cancer Chemother. Pharmacol. 78 (1), 13–26. 10.1007/s00280-016-3037-3 27118574

[B43] Zhou A. P. Bai Y. Song Y. Luo H. Ren X. B. Wang X. (2019). Anlotinib versus Sunitinib as First‐Line Treatment for Metastatic Renal Cell Carcinoma: A Randomized Phase II Clinical Trial. Oncol. 24 (8), e702–e708. 10.1634/theoncologist.2018-0839 PMC6693716 30902918

